# Growth Differentiation Factor 15 in Children with Chronic Kidney Disease and after Renal Transplantation

**DOI:** 10.1155/2020/6162892

**Published:** 2020-02-06

**Authors:** Hjordis Thorsteinsdottir, Cathrin Lytomt Salvador, Geir Mjøen, Anine Lie, Meryam Sugulle, Camilla Tøndel, Atle Brun, Runar Almaas, Anna Bjerre

**Affiliations:** ^1^Division of Paediatric and Adolescent Medicine, Oslo University Hospital, Norway; ^2^Institute of Clinical Medicine, University of Oslo, Norway; ^3^Department of Pediatric Research, Oslo University Hospital, Norway; ^4^Department of Medical Biochemistry, Oslo University Hospital, Norway; ^5^Department of Nephrology, Oslo University Hospital, Norway; ^6^Division of Gynaecology and Obstetrics, Oslo University Hospital, Norway; ^7^Department of Paediatrics, Haukeland University Hospital, Bergen, Norway; ^8^Department of Clinical Medicine, University of Bergen, Norway; ^9^Laboratory for Clinical Biochemistry, Haukeland University Hospital, Bergen, Norway; ^10^Department of Clinical Science, University of Bergen, Norway

## Abstract

Growth differentiation factor 15 (GDF-15) is strongly associated with cardiovascular disease (CVD). The aim of our study was to evaluate plasma and urinary levels of GDF-15 after pediatric renal transplantation (Rtx) and in children with chronic kidney disease (CKD) and its associations to cardiovascular risk factors. In this cross-sectional study, GDF-15 was measured in plasma and urine from 53 children with a renal transplant and 83 children with CKD and related to cardiovascular risk factors (hypertension, obesity, and cholesterol) and kidney function. Forty healthy children served as a control group. Plasma levels of GDF-15 (median and range) for a Tx (transplantation) cohort, CKD cohort, and healthy controls were, respectively, 865 ng/L (463-3039 ng/L), 508 ng/L (183-3279 ng/L), and 390 ng/L (306-657 ng/L). The CKD and Tx cohorts both had significantly higher GDF-15 levels than the control group (*p* < 0.001). Univariate associations between GDF-15 and hyperuricemia (*p* < 0.001), elevated triglycerides (*p* = 0.028), low HDL (*p* = 0.038), and obesity (*p* = 0.028) were found. However, mGFR (*p* < 0.001) and hemoglobin (*p* < 0.001) were the only significant predictors of GDF-15 in an adjusted analysis. Urinary GDF-15/creatinine ratios were 448 ng/mmol (74–5013 ng/mmol) and 540 ng/mmol (5–14960 ng/mmol) in the Tx cohort and CKD cohort, respectively. In the CKD cohort, it was weakly correlated to mGFR (*r* = −0.343, *p* = 0.002). Plasma levels of GDF-15 are elevated in children with CKD and after Rtx. The levels were not associated with traditional cardiovascular risk factors but strongly associated with renal function.

## 1. Introduction

Growth differentiation factor 15 (GDF-15), also known as macrophage inhibitory cytokine-1 (MIC-1), is a distant member of the transforming growth factor-*β* (TGF-*β*) superfamily. It was originally identified by Bootcov et al. in 1997 as one of the macrophages' regulating factors [1]. The placenta is the only tissue that expresses large amounts of the protein under physiological conditions [2], but its expression is upregulated in various pathological conditions. Elevated levels of GDF-15 are strongly associated with cardiovascular disease (CVD) [3], and in large cohorts, GDF-15 has been shown to be an independent predictor of all-cause mortality when adjusted for cardiovascular risk factors, CVD, and other biomarkers [4, 5]. GDF-15 seems to have both protective and adverse effects depending on the state of the cells and the microenvironment [6].

To our knowledge, only one study is published on GDF-15 after renal transplantation (Rtx) in adults [7]. In that study, GDF-15 was related to anemia and hepcidin, indicating its involvement in the pathogenesis of anemia. In addition, GDF-15 was related to creatinine and estimated glomerular filtration rate (eGFR). Urinary GDF-15 levels have also been shown to be elevated and negatively correlated with eGFR in adults with diabetes [8]. Increasing data exists on GDF-15 in children, but only one study on children with kidney disease is published and demonstrates elevated GDF-15 levels in patients on hemodialysis and peritoneal dialysis [9].

We hypothesized that circulating GDF-15 is associated with cardiovascular risk factors in children after Rtx and that plasma and urinary GDF-15 could be used as a biomarker of CVD risk in children. We also wanted to adjust the relation between GDF-15 and CVD risk factors for kidney function as adult studies have indicated a relation between kidney function and GDF-15.

## 2. Material and Methods

### 2.1. Patient Cohorts

*Tx cohort:* children and adolescents ≤ 18 years of age who underwent Rtx at Oslo University Hospital between 2000 and 2015. The patients participated in the HENT (Health after Kidney Transplantation) study and patients were enrolled in 2015-16. Inclusion criteria for the HENT study were a functioning graft for at least 1 year and no ongoing signs of rejection.

*CKD cohort:* children and adolescents < 18 years of age with CKD were included in a cross-sectional study, evaluating biomarkers in CKD and different methods of measuring glomerular filtration rate (mGFR). The children were in a stable phase of their CKD and enrolled at the pediatric departments at Oslo University Hospital and Haukeland University Hospital [10, 11].

Written informed consent was obtained from patients and/or their parents prior to start of the study. The study protocols were approved by the Regional Committee for Medical and Health Research Ethics (references 2009/1008 and 2009/741), and the study was carried out according to the Declaration of Helsinki.

### 2.2. Healthy Control Group

Blood samples from a healthy group of fasting children aged 5-8 years were used as the control group for circulating GDF-15 levels. These healthy children, without any sign of CVD or renal disease, were included as part of a longitudinal pregnancy follow-up study of mother and children after pregnancy complications, i.e., preeclampsia and diabetes mellitus (gestational and type 1) [12, 13].

### 2.3. Anthropometrics

Body Mass Index (BMI) was calculated as kg/m^2^. *Z*-scores for weight, height, and BMI were calculated based on the LMS method, using Norwegian references [14], and overweight and obesity was defined according to BMI cut-off limits proposed by the International Obesity Task Force (isoBMI > 25 for overweight and isoBMI > 30 for obesity) [15].

### 2.4. Renal Function

mGFR was measured by using an injection of Omnipaque® (GE Healthcare, Oslo, Norway; i.e., 647 mg iohexol/mL) with blood sampling after 2 and 5 hours as described in a previous publication [10]. In the Tx cohort, 2 mL of Omnipaque® was given to children under 2 years and 5 mL to children over 2 years while the dose was adjusted to the child's weight in the CKD cohort (<10 kg, 1 mL; 10–20 kg, 2 mL; 20–30 kg, 3 mL; 30-40 kg, 4 mL; and >40 kg, 5 mL).

### 2.5. Blood Pressure

Blood pressure was measured using automatic blood pressure monitors. Hypertension was defined as systolic blood pressure (SBP) or diastolic blood pressure (DBP) over the 95^th^ percentile for age, height, and gender and/or use of antihypertensive medication [16].

### 2.6. Biochemistry

Venous blood samples were obtained after an overnight fast. Hemoglobin was measured by photometry (Sysmex XN). Plasma HDL cholesterol, LDL cholesterol, total cholesterol, and uric acid were measured by enzymatic colorimetric methods and plasma triglycerides by an enzymatic photometric method (Cobas® c702, Roche Diagnostics). The following thresholds were used as definition for cardiovascular risk factors: P‐HDL < 40 mg/dL (1.03 mmol/L), P‐LDL > 130 mg/dL (3.36 mmol/L), P‐cholesterol > 200 mg/dL (5.17 mmol/L), and P‐triglycerides > 150 mg/dL (1.7 mmol/L). Uric acid levels were adjusted with age- and gender-specific normal values, and the 95^th^ percentile was used as the cut-off value for the definition of hyperuricemia [17].

### 2.7. GDF-15

In the two study cohorts, plasma GDF-15 was measured in duplicate, after one freeze-thaw cycle (two cycles for the CKD cohort), by a solid phase sandwich enzyme-linked immunosorbent assay (ELISA) with a human GDF-15 Quantikine® ELISA kit (Bio-Techne). Urinary GDF-15 was measured in duplicate by the same GDF-15 Quantikine® ELISA kit and normalized for urine creatinine.

In the control group, GDF-15 was measured in plasma by an immunoradiometric sandwich assay using a polyclonal, affinity chromatography-purified goat antihuman GDF-15 IgG antibody (R&D Systems). The analyses were performed in duplicate at the laboratory where the assay was developed.

According to a recent study [18], there is a good correlation between the two different methods of measuring GDF-15 in plasma.

### 2.8. Statistical Analysis

Data are described as either median and range or geometric means with 95% confidence interval. Natural logarithmic transformations were performed for achieving more normally distributed data due to a positively skewed distribution of plasma GDF-15 levels and urinary GDF-15/creatinine ratio. For two continuous variables, the strength of associations was measured using Pearson or Spearman correlations depending on the distribution of the data. For continuous variables, the difference between two groups was analyzed using a Mann-Whitney Wilcoxon test or a *t*-test depending on the distribution of the data. Multivariate linear regression was chosen for adjusted analysis of associations between LnGDF-15 and potential explanatory variables. All statistical analyses were performed in Statistical Package for Social Sciences (SPSS) version 21.

## 3. Results

### 3.1. Patient Characteristics

Fifty-three children (32 boys, median age 12.2 years, range 2.3–18 years) with a renal transplant were included. The causes of ESRD were congenital anomalies of the kidney and urinary tract (CAKUT) (*n* = 23), hereditary causes (*n* = 13), glomerulonephritis (*n* = 8), acquired (excluding glomerulonephritis) (*n* = 7), and other or unknown etiologies (*n* = 2). The individual GFR measurements were distributed according to different CKD stages in the following way: 5, 17, 30, and 1 patients in CKD stages 1, 2, 3 and 4, respectively. Eighty-three children with CKD (49 boys, median age 10.1 years, range 2.0-17.5 years) were enrolled, 34 from Oslo University Hospital and 49 from Haukeland University Hospital. The distribution according to CKD stages was as follows: 27, 24, 19, and 13 patients in CKD stages 1, 2, 3, and 4–5, respectively. 11% of the Tx patients and 34% of the CKD patients had significant proteinuria (protein/creatinine ratio > 50 mg/mmol). The patients' basal characteristics and demographics are presented in Table 1.

### 3.2. Immunosuppression

The majority of patients in the Tx group received a tacrolimus-based immunosuppression (*n* = 47), combined with mycophenolate (*n* = 29) and prednisolone (*n* = 48, mean daily dose 0.071 mg/kg). CsA was used in seven patients and nine received everolimus (three as a monotherapy with prednisolone, five in combination with a calcineurin inhibitor, and one with mycophenolate). Azathioprine was used by three patients (in combination with a calcineurin inhibitor and prednisolone). In the CKD group, five patients (6%) received immunosuppressive treatment, one tacrolimus and mycophenolate because of previous limbal transplantation and the remaining four received tacrolimus, mycophenolate, and/or prednisolone as a treatment for glomerular diseases (two glomerulonephritides, steroid-resistant nephrotic syndrome, and Henoch-Schonlein purpura).

### 3.3. Plasma GDF-15 Levels in Children with Renal Failure

**Figure 1:**
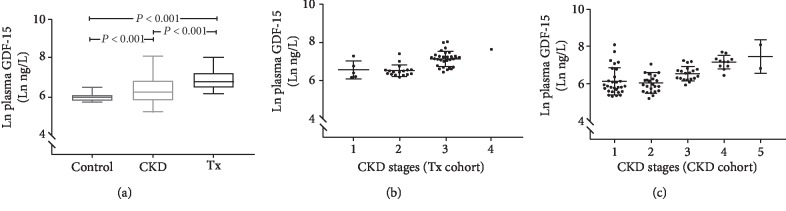
Comparison of plasma GDF-15 levels (mean ± SD) in the Tx cohort, CKD cohort, and healthy controls. Distribution of plasma GDF-15 values (mean ± SD) according to CKD stages in the Tx cohort (b) and CKD cohort (c). Shown in natural logarithmic (Ln) transformation due to skewed distribution.

The respective plasma GDF-15 levels (median and range) for the Tx cohort, CKD cohort, and the control group were 865 ng/L (463-3039 ng/L), 508 ng/L (183-3279 ng/L), and 390 ng/L (306-657 ng/L) (Table 2). As shown in Figure 1, the Tx cohort had significantly higher plasma GDF-15 levels than both the CKD cohort (*p* < 0.001) and the control group (*p* < 0.001). Figure 1 shows as well the distribution of plasma GDF-15 according to the different CKD stages. Plasma GDF-15 levels were also significantly higher in the CKD cohort than the control group (*p* < 0.001). There were no significant differences in plasma GDF-15 levels between genders in either study group.

### 3.4. Plasma GDF-15 Levels and Cardiovascular Risk Factors

23% and 9% of the patients in the Tx cohort had overweight and obesity, respectively. 51% had hypertension and up to 62% had some kind of dyslipidemia (Table 3). In univariate analyses, plasma levels of GDF-15 were significantly higher in obese patients (*p* = 0.028), in patients with high levels of triglycerides (*p* = 0.028), and in patients with low levels of HDL cholesterol (*p* = 0.038). 42% had hyperuricemia and those had significantly higher plasma levels (*p* < 0.001), and uric acid was significantly correlated with plasma GDF-15 (*r* = 0.451, *p* = 0.001) and mGFR (*r* = ‐0.604, *p* < 0.001). For the other cardiovascular risk factors, there were no significant differences in GDF-15 levels. Only one patient had diabetes mellitus type 1 with a slightly elevated HbA1c (7.8%). Hemolytic uremic syndrome (HUS) was the cause of ESRD in this patient, and the patient developed diabetes as a result of pancreas infarcts during the initial presentation of HUS. The rest of the patients in the Tx cohort had normal HbA1c (Table 1).

### 3.5. Plasma GDF-15 Levels and Renal Function

**Figure 2:**
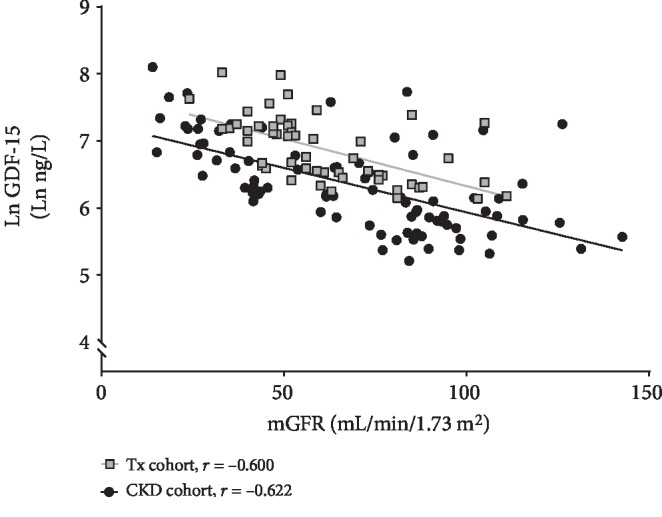
Univariate correlations between plasma GDF-15 and mGFR in Tx and CKD cohorts.

Plasma GDF-15 levels had a significant negative correlation with mGFR in both the Tx cohort (*r* = ‐0.600, *p* < 0.001) and the CKD cohort (*r* = ‐0.622, *p* < 0.001). The distribution is similar in both groups as shown in Figure 2, and the correlation was also significant when the two groups are merged (*r* = ‐0.616, *p* < 0.001). There was no statistically significant difference in mGFR between the Tx cohort and the CKD cohort (*p* = 0.140). Hemoglobin was negatively correlated to plasma GDF-15 levels in both groups and for the two groups combined (*r* = ‐0.580, *p* < 0.001). There were no significant correlations between plasma GDF-15 levels and age in either the two groups separately or the combined group.

In a multivariate model where the two groups were merged for gaining statistical power, mGFR, hemoglobin, and the study group were significant predictors of plasma GDF-15 (Table 4). In a subanalysis for the Tx cohort where cardiovascular risk factors (hypertension, triglycerides, and cholesterol) were taken in as possible explanatory factors in addition to mGFR, age, and sex, mGFR was the only significant predictor (*p* < 0.001). Due to multicollinearity, uric acid was not included in the multivariate analysis.

### 3.6. Urinary GDF-15

There was not a significant difference in urinary GDF-15/creatinine ratio between the Tx cohort and the CKD cohort (Table 2). No significant associations were found with cardiovascular risk factors in either group. In the CKD cohort, there was a significant correlation between urinary GDF-15/creatinine ratio and mGFR (*r* = ‐0.343, *p* = 0.002). In the Tx cohort, urine was only available from 50/53 patients and there was no significant correlation between the GDF-15/creatinine ratio and mGFR in this group (*r* = 0.077, *p* = 0.597). The urinary GDF-15/creatinine ratio correlated positively with plasma GDF-15 levels in the Tx cohort (*r* = 0.408, *p* = 0.003) and the CKD cohort (*r* = 0.422, *p* < 0.001).

## 4. Discussion

In this study, we found that plasma levels of GDF-15 are significantly elevated in children with a renal transplant and in children with chronic kidney diseases compared to healthy children and that plasma GDF-15 levels are strongly associated with kidney function.

To our knowledge, this is the first time GDF-15 has been related to kidney function in a pediatric cohort although an association between renal function and plasma GDF-15 has been found in adults [7, 19]. The knowledge of associations between GDF-15 and renal disease has been increasing. GDF-15 has been suggested as an independent risk factor of mortality in adults with end stage renal disease (ESRD) [19, 20] and for progression of kidney disease [21]. Elevated circulating GDF-15 has been related to incident kidney disease, and it is suggested that it might be useful in predicting the progression of chronic kidney disease, years before clinical onset of the disease [22]. Studies in healthy males and in adults with diabetic nephropathy have shown a faster decline of GFR in patients with high levels of GDF-15 [19, 23]. The role of GDF-15 in decreasing renal function is poorly understood, but in murine models, GDF-15 plays a significant role in the proliferation of acid-secreting intercalated cells in the collecting duct [24] and is an early mediator after induced kidney injury [25].

GDF-15 is a member of the TGF-*β* superfamily, and TGF-*β* is a mediator of fibrosis and inflammation [26]. TGF-*β* is an important profibrotic factor in the kidneys and plays a role in endothelial-to-mesenchymal transition that is suggested to be important in chronic allograft tubular atrophy/interstitial fibrosis [27]. GDF-15 has also been associated with fibrosis in diseases of other organ systems such as dilated cardiomyopathy [28], systemic sclerosis [29], and chronic liver disease [30]. Two recent studies have shown GDF-15 to be associated with biopsy-proven fibrosis in the kidneys, the first in patients with IgA nephropathy [31] and the other in idiopathic membranous nephropathy [32]. GDF-15 might therefore also be a marker or a causative factor of kidney fibrosis that is responsible for decreased renal function in our pediatric cohorts.

Nair et al. found a significant correlation between intrarenal tubulointerstitial expression of GDF-15 mRNA and circulating GDF-15 in 24 patients with CKD [21] which implies that it is produced in the kidneys and might have a pathophysiological role in the progression of CKD and/or the development of interstitial fibrosis. If it is excreted in urine, it could be a valuable, noninvasive marker of kidney function or kidney fibrosis. We found, however, only a weak correlation between urinary GDF-15 and renal function in our CKD cohort and no significant correlation in the Tx cohort. We therefore cannot postulate urinary GDF-15 as a biomarker of either renal function or renal fibrosis. Due to the correlation between GDF-15 and renal function in the present study and strong associations between circulating GDF-15 and fibrosis in other organ systems, we consider the relationship between GDF-15 (urinary and circulating) and renal fibrosis to be worth further exploration.

Serum GDF-15 levels are elevated in disorders of ineffective erythropoiesis such as thalassemia [33], and GDF-15 is a possible mediator of anemia through hepcidin in adult renal transplant recipients [7]. Hepcidin plays an important role in iron metabolism as it negatively regulates plasma iron levels by binding to ferroportin which induces internalization of iron into the reticuloendothelial system. Hepcidin levels are elevated in kidney failure due to decreased renal clearance and inflammatory upregulation which results in reduced availability of plasma iron and anemia [34]. We found strong correlations between plasma GDF-15 and hemoglobin that supports the relationship of GDF-15 to erythropoiesis, but hepcidin levels were not measured in our patients. Hemoglobin and mGFR are interrelated in CKD and our study revealed hemoglobin and mGFR to be equally strong predictors of plasma GDF-15, but this cross-sectional study does not allow us to determine the causal factor in this relationship.

In the adult population, plasma GDF-15 has been associated with progression and prognosis of CVD [3] and may be a potential tool for risk stratification of CVD [35]. We therefore hypothesized that it would be associated to cardiovascular risk factors in our Tx cohort. The prevalence of cardiovascular risk factors in our group of renal transplanted children is high, and we found significant univariate associations between GDF-15, hyperuricemia, elevated triglycerides, low HDL, and obesity. We found, however, that renal function is a major determinant of plasma GDF-15 in children with reduced kidney function. mGFR and hemoglobin were the only significant predictors of GDF-15 in adjusted analysis. Thus, we conclude that while plasma GDF-15 is associated (in unadjusted analyses) with cardiovascular risk factors in renal transplanted children, it is not useful as a biomarker for cardiovascular disease in this group because of the very strong association with renal function.

There are some limitations to our study. Plasma GDF-15 was measured by a different method in the healthy control group. There has however been published a study that compares different methods to measure GDF-15, and it shows a good correlation between the two methods [18]. We are therefore confident that the comparison is reliable. In addition, the study groups are small and heterogeneous with regard to age and underlying diseases. A small sample increases the probability of a type 2 error, but when we have a significant finding, this is less relevant. On the other hand is the Tx group representative for the whole Norwegian population as patients were recruited from the whole country with a high participation rate.

In conclusion, circulating GDF-15 levels are elevated in children after kidney transplantation and in children with decreased renal function. While we found significant univariate associations between GDF-15 and risk factors for CVD as elevated triglycerides, low HDL and obesity, mGFR, and hemoglobin were the only significant predictors of GDF-15 in an adjusted analysis. We found that GDF-15 is associated with renal function in children, and this strong association does not make plasma GDF-15 a useful biomarker for CVD in this population. Whether GDF-15 might be useful in evaluation of kidney fibrosis should be evaluated further. Evaluation of fibrosis in transplant biopsies and possible associations with circulating GDF-15 could be a field of future research at centers where routine surveillance biopsies are performed.

## Figures and Tables

**Table 1:** Basal characteristics of the two study cohorts and the control group. Values in median and range.

	Tx cohort	CKD cohort	Healthy controls
*N*	53	83	40
Age (years)	12.2 (2.3–18.0)	10.1 (2.0–17.5)	6.7 (4.8–8)
Male (*n*, %)	32 (60%)	49 (59%)	
Weight (kg)	39.3 (11.1–90.4)	30.8 (8.96–84.6)	
Weight *Z*-score	-0.45 (-2.60–3.10)	-0.31 (-3.43–2.66)	
Height (cm)	142 (83–184)	137 (74–177)	
Height *Z*-score	-1.52 (-4.4–0.5)	-0.53 (-4.63–2.04)	
BMI (kg/m^2^)	17.9 (14.2–35.4)	17.0 (12.7–33.2)	
BMI *Z*-score	0.34 (-1.49–2.97)	0.20 (-3.30–2.75)	
Overweight/obesity (*n*, %)	12/5 (23/9)	11/3 (13/4)	
Age at Rtx1 (years)	4.4 (0.8–15.8)	—	
Time from Rtx1 (years)	5.0 (1.0–15.5)	—	
Preemptive Rtx1 (*n*, %)	25 (47%)	—	
Total dialysis (months)	9.5 (0.25–39.5)	—	
Rtx1/Rtx2	51/2	—	
LD/DD (*n*, %)	48/5 (91%)	—	
mGFR (mL/min/1.73 m^2^)^a^	56 (24–111)	73 (14–143)	
Hemoglobin (g/dL)	12.2 (7.1–14.8)	12.5 (8.7–15.5)	
HbA1c (%)	5.2 (4.2–7.8)	—	
Protein/creatinine ratio (mg/mmol)	16 (6–193)	27 (3–1084)	
<15 mg/mmol (*n*, %)	24 (46%)	26 (31%)	
15–50 mg/mmol (*n*, %)	22 (42%)	29 (35%)	
>50 mg/mmol (*n*, %)	6 (11%)	28 (34%)	
Etiology of ESRD/CKD			
CAKUT	23 (43%)	27 (33%)	
Hereditary	13 (25%)	23 (28%)	
Glomerulonephritis	8 (15%)	9 (11%)	
Acquired	7 (13%)	10 (12%)	
Vesiculoureter reflux	—	7 (8%)	
Miscellaneous/unknown	2 (4%)	7 (8%)	

^a^For two patients in the Tx cohort, the mGFR is missing because of low GFR, replaced with eGFR.

**Table 2:** Plasma and urinary levels of GDF-15 in the two study cohorts (median and range).

	Tx cohort	CKD cohort	Healthy controls
Plasma GDF-15 (ng/L)	865 (463–3039)	508 (183–3279)	390 (306–657)
Urinary GDF-15 (ng/L)	2740 (449–9183)	2263 (41–28760)	NA
Urinary GDF-15/creatinine ratio (ng/mmol)	448 (74–5013)	540 (5–14960)	NA

**Table 3:** Prevalence of cardiovascular risk factors in the Tx cohort and univariate relations to GDF-15.

		*N* (%)	Geometric mean (ng/L)	95% CI	*p* value
Weight	Normal weight	36 (68%)	937	796-1103	0.028^∗^ ANOVA
	Overweight	12 (23%)	857	639-1150	
	Obesity	5 (9%)	1647	1288-2107	

Blood pressure	Hypertension	27 (49%)	967	799-1170	0.981
	No hypertension	26 (51%)	970	794-1186	

HDL	<40 mg/dL	11 (21%)	1277	889-1832	0.038^∗^
	≥40 mg/dL	41 (79%)	907	786-1047	

LDL	>130 mg/dL	5 (9%)	1103	667-1825	0.536
	≤130 mg/dL	48 (91%)	956	828-1103	

Cholesterol	>200 mg/dL	9 (17%)	952	821-1104	0.462
	≤200 mg/dL	43 (83%)	1088	718-1646	

TG	>150 mg/dL	33 (62%)	1085	914-1288	0.028^∗^
	≤150 mg/dL	20 (38%)	803	655-986	

Uric acid	Hyperuricemia	22 (42%)	1288	668-938	<0.001^∗^
	No hyperuricemia	31 (58%)	792	1096-1512	

^∗^Two-sided *p* value less than 0.05.

**Table 4:** Multiple linear regression model for plasma GDF-15 (Tx and CKD cohorts).

Dependent variable GDF-15			
Risk factor	Unstandardized B	*p* value	95% CI for B
Age (years)	0.005	0.613	(-0.015, 0.026)
Sex	0.085	0.286	(-0.072, 0.243)
mGFR (mL/min/1.73 m^2^)	-0.009	<0.001	(-0.012, -0.006)
Hemoglobin (g/dL)	-0.162	<0.001	(-0.219, -0.115)
Hypertension	0.004	0.956	(-0.152, 0.161)
BMI *Z*-score	-0.096	0.445	(-0.344, 0.152)
Height *Z*-score	-0.146	0.120	(-0.330, 0.036)
Weight *Z*-score	0.113	0.454	(-0.185, 0.411)
CKD vs. Tx	0.262	0.005	(0.008, 0.445)

## Data Availability

The data used to support the findings of this study are available from the corresponding author upon request.
